# Bioprospecting desert plant *Bacillus* endophytic strains for their potential to enhance plant stress tolerance

**DOI:** 10.1038/s41598-019-54685-y

**Published:** 2019-12-03

**Authors:** Ameerah Bokhari, Magbubah Essack, Feras F. Lafi, Cristina Andres-Barrao, Rewaa Jalal, Soha Alamoudi, Rozaimi Razali, Hanin Alzubaidy, Kausar H. Shah, Shahid Siddique, Vladimir B. Bajic, Heribert Hirt, Maged M. Saad

**Affiliations:** 1 King Abdullah University of Science and Technology (KAUST), Center for Desert Agriculture, Thuwal, 23955-6900 Kingdom of Saudi Arabia; 2 King Abdullah University of Science and Technology (KAUST), Computational Bioscience Research Center (CBRC), Thuwal, 23955-6900 Kingdom of Saudi Arabia; 3 King Abdulaziz University, Science and Arts College, Department of Biology, Rabigh, 21589 Kingdom of Saudi Arabia; 4 Bahauddin Zakariya University, Institute of Pure and Applied Biology, Multan, 60800 Pakistan; 5 UC Davis, Department of Entomology and Nematology, One Shields Avenue, Davis, USA; 6 Max F. Perutz Laboratories, University of Vienna, Dr. Bohrgasse 9, 1030 Vienna, Austria; 7 Present Address: Exploration and Petroleum Engineering Center - Advanced Research Center (EXPEC ARC), Saudi Aramco, Dhahran Saudi Arabia; 8 Present Address: Zayed University, College of Natural and Health Sciences, Abu-Dhabi, 144534 United Arab Emirates; 9 University of Jeddah, P-O-BOX No.80327, Jeddah, 21589 Saudi Arabia

**Keywords:** Bacterial host response, Applied microbiology

## Abstract

Plant growth-promoting bacteria (PGPB) are known to increase plant tolerance to several abiotic stresses, specifically those from dry and salty environments. In this study, we examined the endophyte bacterial community of five plant species growing in the Thar desert of Pakistan. Among a total of 368 culturable isolates, 58 *Bacillus* strains were identified from which the 16 most divergent strains were characterized for salt and heat stress resilience as well as antimicrobial and plant growth-promoting (PGP) activities. When the 16 *Bacillus* strains were tested on the non-host plant *Arabidopsis thaliana*, *B. cereus* PK6-15, *B. subtilis* PK5-26 and *B. circulans* PK3-109 significantly enhanced plant growth under salt stress conditions, doubling fresh weight levels when compared to uninoculated plants. *B. circulans* PK3-15 and PK3-109 did not promote plant growth under normal conditions, but increased plant fresh weight by more than 50% when compared to uninoculated plants under salt stress conditions, suggesting that these salt tolerant *Bacillus* strains exhibit PGP traits only in the presence of salt. Our data indicate that the collection of 58 plant endophytic *Bacillus* strains represents an important genomic resource to decipher plant growth promotion at the molecular level.

## Introduction

Plants are largely considered to be meta-organisms due to their dependence on plant-specific growth-promoting bacterial communities^1,2^. This dependence is associated with bacteria that can: 1/increase plant nutrient uptake by nitrogen fixation, phosphate and zinc solubilization and siderophore production, 2/produce phytohormones e.g. indole-acetic acid (IAA), salicylic acid (SA), and abscisic acid (ABA), 3/suppress biotic stressors by production of antimicrobial compounds against plant pathogenic bacteria or fungus, and 4/confer plant tolerance to abiotic stresses such as drought, salinity and extreme temperatures^3–8^. These characteristics led to several attempts to identify plant growth-promoting bacteria (PGPB) that can improve crop growth and yield^9–12^. One possibility is to use biofertilizers to replace or reduce the use of chemical fertilizers that require non-sustainable petroleum sources. Based on Mahdi *et al*.^13^, the demand for chemical fertilizers will exceed the supply by more than 7 million tons by 2020. This shortage of fossil fuels used to produce chemical fertilizers is expected to increase the price of chemical fertilizers and consequently limit crop production worldwide and more so in developing countries. Moreover, chemical fertilizers negatively impact the agro-ecosystem and the environment as nitrogen fertilizers are made from ammonia and their continuous application results in pollution of water through leaching and emission of ammonia gas^14^. Also, phosphate fertilizers, that are imperative to crop production, have limited efficacy as up to 80% of the applied phosphorous precipitates in the soil^14,15^. Conversely, using PGPB as a biofertilizers, improve the availability of phosphorous and nutrient supplies to crops, improve soil structure and promotes a healthy, fertile soil^9–12^.

As sessile organisms, plants are exposed to different abiotic stresses that frequently co-occur such as high temperatures, drought and salinity^16–18^ and exerts devastating effect on crop production^16,17^. In this regard, at present, ∼20% of total cultivated and 33% of irrigated agricultural lands are affected by salt stress^18^ and more than 50% of arable land is expected to be affected by both drought and salinity by 2050^19,20^. Both of these stresses have been shown to affect the water potential and turgor of plants, thereby resulting in a reduction of plant growth^21,22^.

Interestingly, abiotic stresses such as drought and high temperatures also influence the spread of pathogens and insects^23–25^. Thus, products that curb the loss of crops caused by pathogens and diseases are of great interest. In this regard, several studies focused on siderophore producing bacteria as an ecological solution to help plants to tolerate both biotic and abiotic stresses. For example, pyoverdine siderophores produced by Pseudomonads control wilt disease in potato caused by *Fusarium oxysporum*^26^, the inhibition of plant growth caused by *Gaeumannomyces graminis*^27^, as well as the suppression of maize root diseases caused by *Macrophomina phaseolina, F. moniliforme* and *F. graminearum*^28^. Ruiz *et al*.^29^ also demonstrated that the plant disease suppressing *Pseudomonas protegens* strain survives in a toxic environment created by the metal chelating mycotoxin Fusaric acid (produced by *Fusarium* strains) by producing metal scavenging siderophores including pyoverdine and pyochelin. Butaite *et al*.^30^ recently demonstrated that non-producers of siderophores, with the appropriate siderophore-receptors, can exploit foreign siderophores, while siderophore-producers that generate exclusive siderophore types render iron acquisition inaccessible to competing strains that lack the appropriate receptor.

Several studies tried to identify appropriate biofertilizers focusing on bacteria belonging to *Bacillus*^31–33^, *Azospirillum*^34^, *Pseudomonas*^34^, *Rhizobium*^34^, *Ralstonia*^35^, *Burkholderia*^35^, and *Klebsiella*^35^ genera that can alleviate these stresses for several crops such as rice^36,37^, maize^38,39^, tomato^40^ and wheat^41^. Not all PGPB can be commercialized as their resilience to different environmental stresses are low. In contrast, PGPB belonging to the *Bacillus* and *Pseudomonas* genera are suitable biofertilizers, as they can survive in diverse biotic and abiotic environments^42–46^. Moreover, *Bacillus*-based biofertilizers display high resilience to diverse environmental stresses due to their spore-forming ability. They also produce metabolites that confer to *Bacillus* strains better biofertilizer qualities than the non-spore forming *Pseudomonas* strains^47^. Some examples of *Bacillus*-based biofertilizers include Alinit^48^, Kodiak (*Bacillus subtilis* GB03)^49^, Quantum-400 (*B. subtilis* GB03), Rhizovital (*B. amyloliquefaciens* FZB42)^50,51^, and YIB (*Bacillus* spp.)^52^. Currently, none of these commercialized biofertilizers confers tolerance to crops against salt stress, or salt and drought stress simultaneously. These factors highlight the urgency for developing biofertilizers that can confer crop resilience to multiple biotic and abiotic stresses. In addition, very few studies focused on the identification of desert plant endophytic *Bacillus* that can alleviate salt stress as biofertilizers.

In the present study, we examined the endophytic bacterial community in plants growing in the Thar desert of Pakistan. We screened *Bacillus* endophytes for their ability to: 1/support plant growth, 2/fix nitrogen, solubilize phosphate, 3/provide protection against biotic stresses, and 4/confer tolerance to salt stress. This search for potential *Bacillus* biofertilizers was performed as part of the DARWIN21 project (www.darwin21.org).

## Results and Discussion

### Isolation and characterization of root endophytes from diverse desert plant species

We collected our samples from the Thar Desert region in Pakistan. Table S1 describes the characteristics of the sampling site and chemical properties of the soil. Briefly, the sampling site had sandy nutrient-poor soil with less than 5% water content and a pH of 7.31. The physicochemical properties and weather conditions were very comparable with different desert regions in the Arabic peninsula^53^.

**Figure 1:**
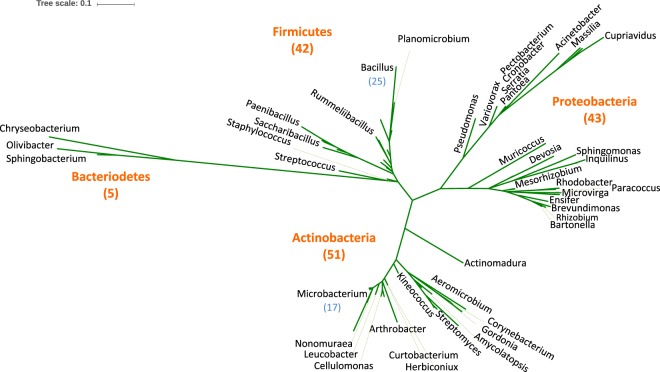
Un-rooted phylogenetic tree based on 16S rRNA gene sequence showing the diversity of bacteria isolated from all plants, at both the phylum and genus level. The numbers in parentheses represent the total count of unique isolates at each level.

**Figure 2:**
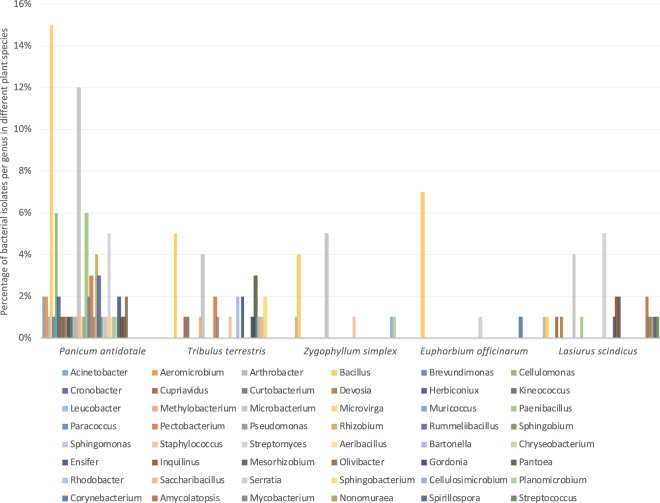
Bar graph showing the distribution and diversity of bacteria cultivated from each plant at genus level. Genera represented by one isolate are not shown.

Bacteria were isolated from a total of five plant species: 1/two Z*ygophyllum simplex*, 2/three *Panicum antidotale*, 3/two *Tribulus terrestris* and 4/one *Euphorbium officinarum* and *Lasiurus scindicus* plants. From surface-sterilized roots, a total of 368 bacterial endophytes were isolated from the five plant species (Table S2). Based on 16S rRNA gene sequences, a taxonomic affiliation of the bacterial isolates was assigned to 141 of the 368 isolates with 99% 16S rRNA gene sequence identity, however the rest of the isolates (227 isolates) were excluded from the current analysis due to redundancy of the identification on the genius/species level or low sequence identity in database. These 141 strains belong to four major phyla: Firmicutes (42 strains; 8 genera), Actinobacteria (51 isolates; 17 genera), Proteobacteria (43 strains; 24 genera) and Bacteroidetes (5 strains; 3 genera) (Fig. 1). Using only few plant individuals (1–3 plants) per species may skew the bacterial diversity as there is no guarantee that the plants used in this study reflect the general bacterial diversity found in the plant species at that location and larger sample sizes might affect this distribution (Fig. 2).

Most of the endophyte strains analyzed in the present work showed a dominance of *Bacillus* of the five plant species from the Thar Desert: 17% of the 141 characterized strains belonged to *Bacillus* (Firmicutes) as the dominant taxon, followed by the Actinobacteria genus *Microbacterium* (12%). Moreover, only *Bacillus* endophytes were isolated from all plant types (Fig. 2). Interestingly, a recent report by Eida *et al*.^53^, showed a similar pattern, as *Bacillus* was found to be one of the most dominant genera in a number of desert plants. *Bacillus* was representing a 33% of the total bacteria isolated from *E. granulata*, 22% from *T. terrestris*, 5% in *P. turgidum* and 9% in *Z. simplex*.^53^.

It is interesting to note that Koberl *et al*.^54^ showed that bacteria isolated from Adleya desert soil in Egypt were similarly dominated by Actinobacteria and Proteobacteria, but the Firmicutes numbers were less than half that of Actinobacteria. Marasco *et al*.^55^ further reported that bacteria isolated from the endosphere, rhizosphere, and root-surrounding soil from desert farmed *Capsicum annum* L. plants (growing in the desert region in Egypt, near El-Tawheed) were primarily Proteobacteria and Firmicutes, while non-cultivated arid soil harbored more Actinobacteria (genus *Cellulosimicrobium*) and Firmicutes (genus *Bacillus*) than Proteobacteria. This result was corroborated by Dai *et al*.^56^ in part, who reported the dominance of Proteobacteria and Firmicutes endophytes in *Carragana microphylla* growing in a plantation in the desert-region of Ningxia Hui, China. Several other studies similarly reported the dominance of Firmicutes as endophytes in plants growing in desert or arid saline land^57–59^. Taken together, the results from our study and the other endorhizosphere studies suggest that Firmicutes (especially the genus *Bacillus*) counts become relatively more abundant with host interaction.

**Figure 3:**
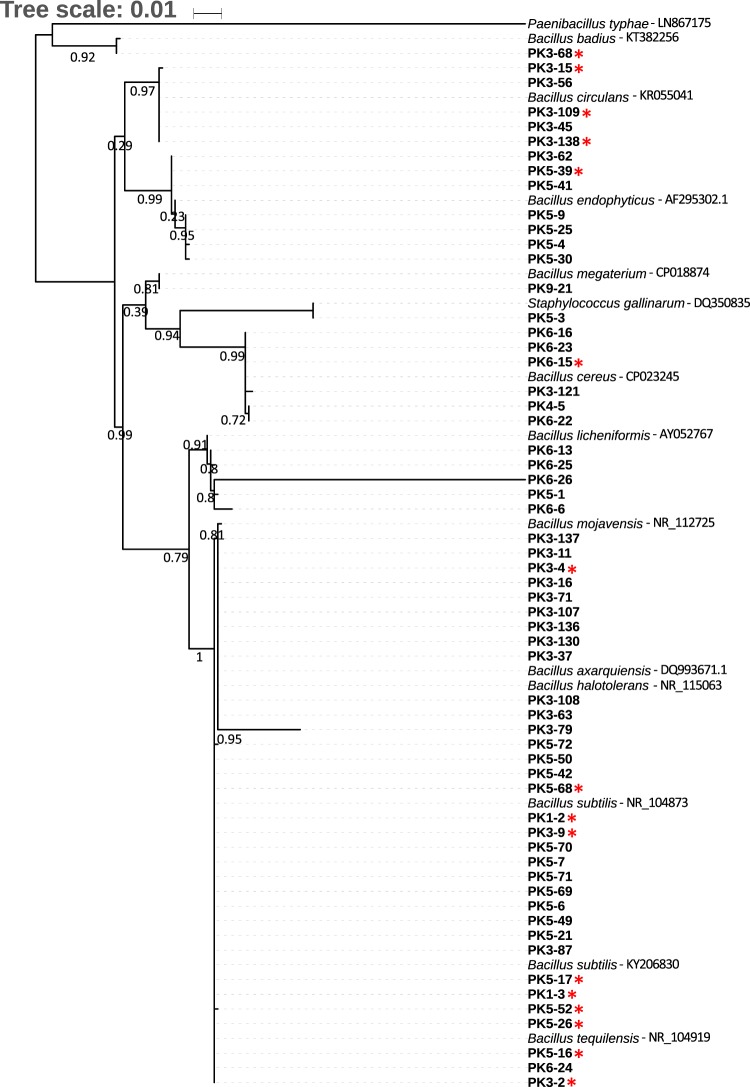
Phylogenetic tree showing the relationship of the 58 newly isolated *Bacillus* strains, based on their 16S rRNA gene sequences. The strains used in this screen are marked with an asterisk (*). *P. typhae* [LN867175] was used as an out-group for the phylogenetic tree. *Bacillus* type strains used to construct the tree, include *B. subtilis* [KY206830], *B. subtilis* [NR_104873], *B. badius* [KT382256], *B. circulans* [KR055041], *B. endophyticus* [AF295302.1], *B. megaterium* [CP018874], *B. cereus* [CP023245], *B. licheniformis* [AY052767], *B. mojavensis* [NR_112725], *B. axarquiensis* [DQ993671.1], *B. halotolerans* [NR_115063], *B. tequilensis* [NR_104919], and *Staphylococcus gallinarum* [DQ350835]. Branches with support less than 50% were collapsed. Bootstrap values are shown in the tree branches.

As *Bacillus* diversity at the species level was more pronounced than that for *Microbacterium*, we focused our search for PGPB on the 58 isolates belonging to the *Bacillus* genus. The 16S rRNA gene sequences of the 58 isolates were used to construct a phylogenetic tree (Fig. 3), which categorized the strains into 8 clusters. Strains from each cluster were selected and tested for their ability to grow in liquid media. Some strains were unable to grow in liquid media, thus only 16 strains were selected for further screening for plant growth-promoting **(**PGP) effects.

### Identification of potential Bacillus PGPB

#### Screening of Bacillus strains for common PGP traits

The capacity to fix nitrogen, solubilize phosphate or produce indole-3-acetic acid (IAA) are key traits of PGPB^10–12^. Zinc can also be a limiting factor in plant growth that can be improved by PGPB^34,35^. We therefore screened the 16 *Bacillus* strains for their ability to solubilize phosphorous (P) and zinc oxide (ZnO) or produce (IAA), and ammonia (Table 1). Among these, only *B. subtilis* PK5-26 and *B. cereus* PK6-15 could solubilize P and ZnO, respectively. Also, *B. badius* PK3-68 and the *B. circulans* strains PK3-15, PK3-138 and PK3-109 were the only bacteria capable of producing IAA. However, most of the strains (13 out of 16) were capable of producing ammonia. *B. subtilis* PK5-26, *B. cereus* PK6-15 and *B. badius* PK3-68, that exhibited some of the other PGP traits (such as solubilizing P and ZnO, and production of IAA), were amongst the strains capable of producing ammonia. Such PGP traits have been reported for other *B. cereus*^60,61^, *B. subtilis*^31–33^ and *B. circulans*^62^ strains, but, to our knowledge, our study is the first to report a *B. badius* strain with PGP traits. Studies related to zinc solubilizing bacteria (ZSB) are less common^63^, even though this trait has been shown to positively facilitate plant growth. Nonetheless, the search for ZSB is on the rise, and a zinc-solubilizing *Bacillus* strain recovered from soybean rhizosphere soil significantly increased the Zn content in soybean seeds when compared with uninoculated controls^64^.

**Table 1:** *Bacillus* strains screened for plant growth promotion traits. In this table, potential biocontrol agents against, 1/*Pseudomonas syringae* DC3000 are indicated by *, 2/*Botrytis cinerea* is indicated by √, and 3/*Alternaria brassicicola* is indicated by ∞.

Strain code	Identification based on 16S rRNA sequencing	Nutrient uptake traits		Growth-promoting traits		Disease suppression traits	
		Phosphate solubilization	Zinc solubilization	IAA Production	Ammonia production	Siderophore production	Anti-microbial effects
PK3-68	*Bacillus badius*	(−)	(−)	(+)	(+)	(−)	(∞)
PK3-9	*Bacillus subtilis*	(−)	(−)	(−)	(+)	(+)	(∞)
PK3-2	*Bacillus tequilensis*	(−)	(−)	(−)	(+)	(−)	(∞)
PK3-15	*Bacillus circulans*	(−)	(−)	(+)	(−)	(−)	(∞)
PK3-138	*Bacillus circulans*	(−)	(−)	(+)	(−)	(−)	(∞)
PK5-26	*Bacillus subtilis*	(+)	(−)	(−)	(+)	(+)	(−)
PK5-39	*Bacillus circulans*	(−)	(−)	(−)	(+)	(−)	(∞)
PK1-2	*Bacillus subtilis*	(−)	(−)	(−)	(+)	(+)	(−)
PK1-3	*Bacillus subtilis PY79*	(−)	(−)	(−)	(+)	(+)	(∞)
PK5-17	*Bacillus subtilis PY79*	(−)	(−)	(−)	(+)	(−)	(∞)
PK5-16	*Bacillus tequilensis*	(−)	(−)	(−)	(+)	(+)	(∞)
PK5-68	*Bacillus subtilis*	(−)	(−)	(−)	(+)	(−)	(−)
PK5-52	*Bacillus subtilis*	(−)	(−)	(−)	(+)	(−)	(∞)
PK6-15	*Bacillus cereus*	(−)	(+)	(−)	(+)	(−)	(√, ∞)
PK3-109	*Bacillus circulans*	(−)	(−)	(+)	(−)	(−)	(*, √)
PK3-4	*Bacillus mojavensis*	(−)	(−)	(−)	(+)	(−)	(∞)

Elsewhere positive activity (formation of cleared zone) is indicated using (+) and negative activity (no cleared zone) is indicated using (−).

#### Screening of Bacillus strains for siderophore production

Several studies reported siderophore functions in scavenging of iron from the host or environment^65^, as well as in the biological control of pathogens, as several siderophores exhibit antimicrobial activity^66,67^. Thus, we also screened the 16 *Bacillus* strains for siderophore production (Table 1). Only the *B. subtilis* strains PK3-9, PK1-2, PK1-3 and PK5-26 and *B. tequilensis* PK5-16 exhibited siderophore production. The siderophores produced by these and most other *Bacillus* strains have generally not been screened for their antimicrobial effects. However, *B. subtilis* CAS15 was shown to produce the siderophore bacillibactin which significantly reduces the incidence of *Fusarium* wilt disease in pepper^31^. Since iron supplementation reduced this biocontrol effect, these results suggest that bacillibactin may be responsible for the biocontrol effect^31^. Beyond this study, the reports by Butaite *et al*. (2017) and others^26–30^ (mentioned in the “Background” section) suggest that iron scavenging and antimicrobial functions of siderophores are both shaping microbiome diversity and community dynamics. Thus, siderophore producing strains such as the *B. subtilis* strains PK3-9, PK1-2, PK1-3 and PK5-26 identified in this study, may be essential components of a biofertilizer consortium.

#### Screening for antimicrobial effects against selected known plant pathogens

Al-Amoudi *et al*.^68,69^ reported that Firmicutes, and specifically strains from the genus *Bacillus*, are better targets for antimicrobial bioprospecting than Actinobacteria due to the selection pressure in environments exposed to high salinity and hydrocarbon contamination. For that reason, we further verified if these 16 *Bacillus* strains have antimicrobial effects that might confer disease resistance to plants. Despite the fact that only five strains showed siderophore production (the small sample size could affect this result), it is still possible that these *Bacillus* strains have antimicrobial activity via other mechanisms^45,70–72^. All strains were screened against the bacterial pathogen *P. syringae* that causes bacterial speck disease^73^, and the fungal pathogens *Botrytis cinerea* and *Alternaria brassicicola* that cause grey mould^74^ and rot disease^75^, respectively. Most strains showed a potential as biocontrol agent except for *B. subtilis* PK1-2, PK5-26 and PK5-68. Specifically, 12 of the 16 strains exhibited antimicrobial effects against *A. brassicicola*. However, only *B. circulans* PK3-109 exhibited antimicrobial effects against *P. syringae*, while *B. cereus* PK6-15 and *B. circulans* PK3-109 exhibited antimicrobial effects against *B. cinerea*. Thus, only *B. subtilis* PK3-9 exhibits siderophore production and antimicrobial effects against *A. brassicicola*. Moreover, *B. circulans* PK3-109 and *B. cereus* PK6-15 were the only strains that exhibit antimicrobial effects against two of the plant pathogens used in this screening process. Also, *B. subtilis* PK1-3, PK5-52 and PK3-9, *B. tequilensis* PK3-2 and *B. circulans* PK3-15 and PK3-138 displayed the most effective clearing of *A. brassicicola*. However, this finding is not surprising as *B*. *subtilis* isolate B7^76^ and *B. subtilis* OTPB1^77^ were reported to have antimicrobial effects against *A. brassicicola*. Here too, it should be noted that the PGP traits are only representative of the 16 strains tested in this study, and do not necessarily reflect the capabilities of the other *Bacillus* strains isolated in this study.

#### Bacillus strain tolerance towards abiotic stresses

The selected 16 strains were also evaluated for their resilience against low (0.5 M NaCl), mild (1 M NaCl), high (1.5 M NaCl) and severe (2 M NaCl) salt stress conditions (see Table 2). All 16 strains grew well on Luria-Bertani (LB) media and LB media + 0.5 M NaCl. However, *B. tequilensis* PK5-16 only grew under low salt stress conditions (0.5 M NaCl), while all other strains could grow under high salt stress conditions (1.5 M NaCl). *B. subtilis* PK5-26, *B. tequilensis* PK3-2 and *B. circulans* PK5-39, were the only isolates able to grow under severe salt stress conditions (2 M NaCl). With the exception of *B. tequilensis* PK5-16, these results suggest that most of the bacteria are halophiles, growing best in media containing 0.5–2.5 M NaCl^78,79^. This characteristic feature makes these bacteria ideal candidates for growing plants in saline areas or upon saline irrigation. The next step is of course to test whether these bacteria can also interact beneficially with crop plants in greenhouse experiments and finally in open agriculture.

**Table 2:** *Bacillus* strain resilience against abiotic stresses. *Bacillus* strains were evaluated for their resilience to heat at multiple temperatures including 28 °C, 37 °C, 42 °C and 50 °C, as well as for their resilience to salinity and at various salt concentrations including 0.5% NaCl, 1% NaCl, 1.5% NaCl, and 2% NaCl at 28 °C.

Strain code	Identification based on 16S rRNA sequencing	Media	Temperature(°C)			NaCl(M)		
			28 & 37	42	50	0.5	1 & 1.5	2
PK3-68	*Bacillus badius*	LB	(+)	(+)	(−)	(+)	(+)	(−)
PK3-9	*Bacillus subtilis*	LB	(+)	(+)	(+)	(+)	(+)	(−)
PK3-2	*Bacillus tequilensis*	LB	(+)	(+)	(+)	(+)	(+)	(+)
PK3-15	*Bacillus circulans*	LB	(+)	(+)	(−)	(+)	(+)	(−)
PK3-138	*Bacillus circulans*	TSA	(+)	(+)	(+)	(+)	(+)	(−)
PK5-26	*Bacillus subtilis*	TSA	(+)	(+)	(+)	(+)	(+)	(+)
PK5-39	*Bacillus circulans*	TSA	(+)	(+)	(+)	(+)	(+)	(+)
PK1-2	*Bacillus subtilis*	TSA	(+)	(+)	(+)	(+)	(+)	(−)
PK1-3	*Bacillus subtilis PY79*	TSA	(+)	(+)	(+)	(+)	(+)	(−)
PK5-17	*Bacillus subtilis PY79*	TY	(+)	(+)	(+)	(+)	(+)	(−)
PK5-16	*Bacillus tequilensis*	TY	(+)	(+)	(+)	(+)	(−)	(−)
PK5-68	*Bacillus subtilis*	TY	(+)	(+)	(+)	(+)	(+)	(−)
PK5-52	*Bacillus subtilis*	TY	(+)	(+)	(+)	(+)	(+)	(−)
PK6-15	*Bacillus cereus*	R2A	(+)	(+)	(−)	(+)	(+)	(−)
PK3-109	*Bacillus circulans*	R2A + 1.5% NaCl	(+)	(+)	(−)	(+)	(+)	(−)
PK3-4	*Bacillus mojavensis*	LB	(+)	(+)	(+)	(+)	(+)	(−)

(+) Indicates growth and (−) no growth.

Since the strains were isolated from the Thar desert, where large temperature differences occur between night and day, the 16 strains were also screened for their thermotolerance (Table 2). All strains grew at 28 °C, 37 °C and 42 °C on LB media. The majority of strains were also able to grow at 50 °C, except *B. badius* PK3-68, *B. circulans* PK3-15 and PK3-109 and *B. cereus* PK6-15. These data suggest that most of the strains may be simple/moderate thermophiles, as they were able to grow under heat stress conditions up to 50 °C.

We shortlisted *Bacillus* strains for further assessment of their ability to confer salt-resilience to model plants. For strain selection, we picked strains that are characterized with one or more PGP traits and with disease suppression capabilities. *Bacillus* strains with PGP traits and no antimicrobial activity were also included. The shortlisted strains were *B. cereus* PK6-15, *B. badius* PK3-68, *B. circulans* PK3-15, PK3-138, PK3-109, *B. subtilis* PK3-9, PK1-2, PK1-3, PK5-26 and *B. circulans* PK5-39.

### Testing *Bacillus* strains for their ability to enhance *Arabidopsis* salt stress tolerance

**Figure 4:**
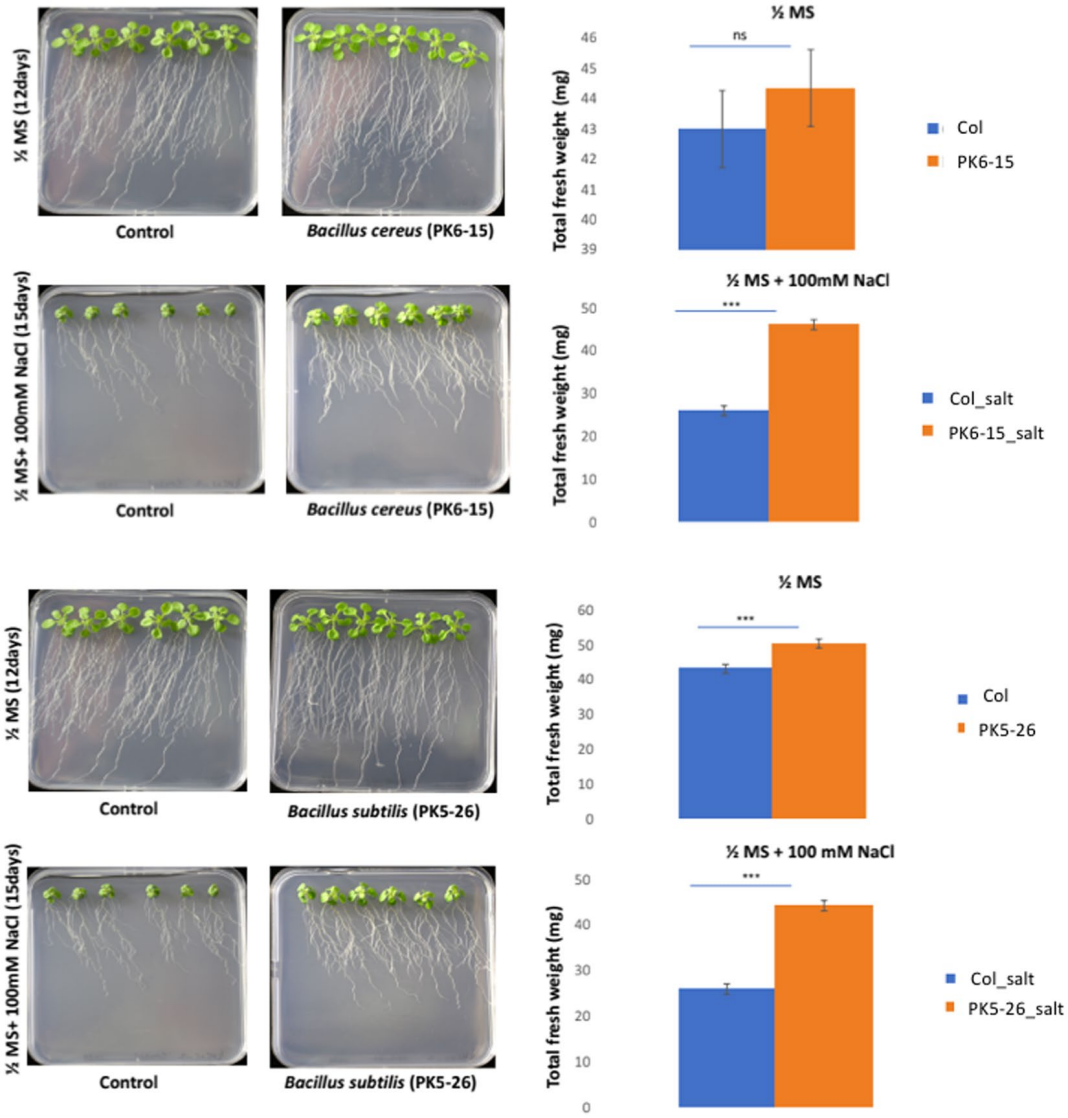
Screening assay of *A. thaliana* inoculated with (**A**) *Bacillus cereus* PK6_15 and (**B**) *Bacillus subtilis* PK5_26 in non-salt (1/2 MS, 12 days) and in salt (1/2 MS + 100 mM NaCl, 15 days) conditions. The total fresh weight of *Arabidopsis* is presented as the mean of three biological experiments. Asterisks indicate statistical differences compared to the control samples under the same conditions based on Mann-Whitney U Test (*P < *0.05*, **P < *0.01*, ***P < *0.001*). Abbreviations: **½ MS:** ½ strength Murashige and Skoog (MS) basal salt macronutrient solution for plant medium; **½ MS** + **100 mM NaCl:** ½ strength Murashige and Skoog (MS) basal salt macronutrient solution with 100 mM NaCl salt added for plant stress medium; **Col:** *Arabidopsis thaliana* Columbia strain (Control).

To test the PGP potential of the *Bacillus* strains on plants, we inoculated *Arabidopsis thaliana* seedlings with strains showing positive PGP traits and disease suppression capabilities. In Table S3, we list the strains based on their ability to increase the fresh weight of *A. thaliana* compared to uninoculated control plants under salt stress conditions (100 mM NaCl). Bacteria were categorized as plant growth-promoting bacteria (+)PGPB and/or salt tolerance plant growth-promoting bacteria (+)ST-PGPB if they confer a statistically significant fresh weight increase to *A. thaliana* when compared to the uninoculated control in the absence of NaCl and in the presence of NaCl, respectively. A *p* value of ≤0.05 was used to determine the statistical significance and is indicated with an asterisk in Table S3. *B. subtilis* PK5-26, *B. cereus* PK6-15, and *B. circulans* PK3-109 (not shown in Fig. 4) significantly enhanced plant growth despite salt stress. When compared to uninoculated control plants, these strains doubled the fresh weight of *A. thaliana* under salt stress conditions (Fig. 4, Table S3). *B. subtilis* PK5-26 slightly increased plant growth under non-salt stress conditions as well as under salt stress conditions. However, *B. circulans* PK5-39 and PK3-138 did not increase the growth of *A. thaliana* under non-salt or salt stress conditions despite their ability to produce ammonia and IAA, respectively. This result indicates that strains displaying PGP traits *in vitro* may not necessarily function as PGPB *in planta*. Interestingly, *B. circulans* PK3-15 and PK3-109, *B. cereus* PK6-15 and *B. subtilis* PK3-9 did not display growth under non-salt conditions, but increased the fresh weight of *A. thaliana* by more than 50% when compared to the uninoculated control plants under salt stress conditions. These results suggest that the presence of NaCl may be a trigger for these bacteria to induce factors that facilitate the growth of *A. thaliana* under salt stress conditions.

At present, the nature of the growth stimulating bacterial factors is unknown. However, a recent study described the desert plant *Indigofera argentea* endophyte *Enterobacter* sp. SA187, to produce 2-keto-4-methylthiobutyric acid (KMBA) to facilitate salt stress tolerance via stimulating the ethylene signaling pathway in *Arabidopsis*^80^. Moreover, SA187 was reported to increase the yield of the forage crop alfalfa when submitted to saline irrigations^80^, providing a proof of concept for the potential use of desert microbes in enhancing the growth of crop plants.

Also, plants adopt different mechanisms to cope with the stress of toxic ions^81^. In Eida *et al*.^82^, we demonstrate that five phylogenetically diverse bacteria isolated from the rhizosphere of three different desert plants induced salinity stress tolerance in *A. thaliana* through similar tissue-specific Na^+^ and K^+^ distribution patterns and transcriptional regulation of ion transporters. Currently, we do not know by which mechanisms the isolated *Bacillus* strains render *Arabidopsis* salt tolerance and further studies at the transcriptome and metabolome level are necessary to define their mode of action.

### Concluding Remarks

Like similar studies, the results on the characterization and activity of the described *Bacillus* isolates of this study are limited by the choice of the plants, sample size and the culture-dependent approach. Nonetheless, this is the first work to identify a zinc-solubilizing *B. cereus* and to report the ability of multiple *Bacillus* strains to promote/increase plant growth under salt stress conditions. Interestingly, several *Bacillus* strains only showed an increase in plant growth in the presence of salt but not in the absence of salt stress. These results suggest that salt stress might trigger the production of plant factors that ultimately stimulate yet unknown bacterial factors to allow plant growth under salt stress conditions. Overall, in this work, we identified several *Bacillus* strains that promote plant growth under ambient and/or salt stress conditions. This work further provides the basis for a genetic evaluation of PGP traits in the context of optimizing agriculture of crops under conditions of saline water irrigation. The combination of PGP traits together with the capability of the selected *Bacillus* strains to enhance plant tolerance to salt stress makes them good candidates for sustainable agriculture. To reach this aim, further evaluation of the PGPR quality of the selected *Bacillus* strains with a variety of crops will be necessary under different culture conditions, including field tests on different soils and saline irrigation. From the variety of the isolated *Bacillus* strains, we hope that some will turn out to protect crops under various abiotic challenges and ensure crop productivity and yield for future generations.

## Materials and Methods

### Study site description

Samples were collected from the Thar desert region in Pakistan (24°45′00.4″69°56′00.8″E) at an altitude of 28 m. The selected location is characterized by low humidity, high evaporation rates, high temperature and limited rainfall. The criteria of plant species collected was based on the plants being indigenous species, perennials woody shrubs/sub-shrubs for easy access to the whole root system and are growing in different desert region including the Arabian Peninsula. Samples consisted of three plant individuals (PK3, PK3b, PK9) from *P. antidotale*, two plant individuals (PK5, PK7) from *T. terrestris*, two plant individuals (PK1, PK6) from *Z. simplex*, and one plant individual from both *E. officinarum* (PK4) and *L. scindicus* (PK8), collected in Zip plastic bags and kept at 4 °C (Bahauddin Zakariya University, Institute of Pure and Applied Biology, Multan, Pakistan) before shipment to Saudi Arabia.

### Soil analysis

Three soil samples were collected from a site (24°45′00.4″N69°56′00.8″E) devoid of vegetation but in close proximity (approximately 2 m away from the vegetation) to the plant samples site. Each soil sample was collected up to 20 cm of depth and placed in sterile tubes that were stored at 4 °C prior to processing. Triplicates of one gram of soil from each sample were used for soil analysis by drying thoroughly followed by nitric acid (1 M) digestion as described in Alzubaidy *et al*.^83^. Element measurement was performed using Inductively Coupled Plasma Optical Emission Spectrometry (Varian 720-ES ICP OES, Australia). Elements measured include P, K, Ca, Cu, Mn, Pb, Na, Ni and S. The instrument settings were: power (KW) 1.2, plasma flow (L/min) 1.65, auxiliary flow 1.5, nebulizer flow (L/min) 0.7, sample uptake delay (L/sec) 70, pump rate (rpm) 15 and rinse time (sec) 35. Carbon and Nitrogen were measured using Flash 2000 (Thermo Scientific) according to^83^. pH was measured using the 5 Star pH Portable Meter (Thermo Scientific, USA). All the samples were measure as three replicated with three to four technical measurement for each replicate. Three biological replicates were measured for all samples.

### Isolation and cultivation of endophytes

As mentioned above, the samples consisted of three plant individuals (PK3, PK3b, PK9) from *P. antidotale*, two plant individuals (PK5, PK7) from *T. terrestris*, two plant individuals (PK1, PK6) from *Z. simplex*, and one plant individual from both *E. officinarum* (PK4) and *L. scindicus* (PK8). Briefly, nine surface sterilized root systems from the nine plant individuals were used for the extraction of endophytic bacteria. The bacterial endophytes were isolated using the standard cultivation-based technique. The root systems from the nine plant individuals were surface sterilized by dipping in 70% ethanol for 30 s, then 2% sodium hypochlorite for 5 minutes, followed by washing with sterilized distilled water. The sterilized roots were then macerated with 0.8% saline solution and subjected to serial dilution (10^−2^–10^−5^). Each dilution was spread in duplicate on the different media (TSA, Tryptone-Yeast (TY), R2A, LB, and R2A with 1.5% NaCl) plates. In addition, 1.5% NaCl was added to R2A was used as a fifth media to enable the isolation of endophytes that can grow in high salt concentrations. Not all colonies growing on the media plates were selected for isolation. Based on colony morphology characteristic e.g. form, elevation, margin, surface, opacity and pigmentation, only bacterial colonies were hand-picked and further purified. Single colonies were isolated from 10^-4^ and 10^-5^ dilution, in total 368 colonies were isolated; 118 from TSA, 37 from TY, 80 from R2A, 52 from LB, and 81 from R2A + 1.5% NaCl.

### Taxonomical identification

Isolated strains were identified through the sequencing of their 16S rRNA genes. Bacterial DNA was extracted using GenElute™ Bacterial Genomic DNA Kits, following manufacturer’s instructions. PCR amplification of 16S rRNA genes was performed using the extracted genomic DNA as the template and the universal primers 27F (5′-AGAGTTTGATCCTGGCTCAG-3′) and 1492R (5′-TACGGYTACCTTGTTAGCACTT-3′). Thermocycler conditions started with initial denaturation at 95 °C for 1 min, followed by 30 cycles at 95 °C for 30 seconds, annealing at 55 °C for 45 seconds and extension at 72 °C for 1.5 minutes, and a final polymerization extension at 72 °C for 5 minutes. PCR amplification was verified by 1% agarose gel electrophoresis and those showing positive signal, were purified using EXOSAP IT (Invitrogen) then sent for Sanger sequencing at Bioscience Core Laboratory, King Abdullah University of Science and Technology (KAUST).

### Phylogenetic analyses

In order to study the evolutionary history and taxonomical relationships among isolated *Bacillus* species, the 16S rRNA gene sequences for 58 newly isolated strains were compared with those in the GenBank database using NCBI BlastN. *P. typhae* [LN867175] was used as an out-group for the phylogenetic tree. *Bacillus* type strains used to construct the tree, include *B. subtilis* [KY206830], *B. subtilis* [NR_104873], *B. badius* [KT382256], *B. circulans* [KR055041], *B. endophyticus* [AF295302.1], *B. megaterium* [CP018874], *B. cereus* [CP023245], *B. licheniformis* [AY052767], *B. mojavensis* [NR_112725], *B. axarquiensis* [DQ993671.1], *B. halotolerans* [NR_115063], *B. tequilensis* [NR_104919], and *Staphylococcus gallinarum* [DQ350835]. The T-Coffee multiple sequence aligner version 11^84^ was used to align the 16S rRNA sequences using the parameter ‘t_coffee -mode rcoffee’. Subsequently, to identify conserved blocks from the multiple sequence alignment (MSA), the Gblocks 0.91b^85^ was applied onto the MSA by using the minimum sequence for flank position at 85%, maximum contig nonconserved position at 8, and minimum block length at 10. Next, we employ PhyML version 20120412^86^, a widely used phylogeny tool based on maximum-likelihood principle. For building the phylogeny, the bootstrap was set to SH-like branch supports, HKY85^87^ was used as the nucleotide-based model and parameter optimization was implemented for the tree topology, branch length and rate parameters. Finally the Newick output from PhyML was used as input for the tree-drawing tool, TreeDyn 198.3^88^ where all branches with branch support values smaller than 50% were collapsed.

### Literature search

A literature search was conducted using PubMed^49^ and PubMed Central^49^ to determine the number of articles that have assessed plant growth promotion through phosphate solubilization, zinc solubilization and indole-3-acetic acid production (see Table 3).

**Table 3:** A literature search was conducted for studies focused on plant growth promotion through PGP traits such as production of indole-3-acetic acid, phosphate-solubilization, zinc-solubilization.

Query	Keywords	PubMed Articles published		PubMed Central Articles published	
		30.06.17	31.12.19	30.06.17	31.12.19
Query 1	(plant growth-promoting OR plant growth promotion) AND (phosphate-solubilizing or phosphate solubilizing)	115	145	627	890
Query 2	(plant growth-promoting OR plant growth promotion) AND (production of indole-3-acetic acid OR produces indole-3-acetic acid OR indole-3-acetic acid biosynthesis)	452	548	2185	2722
Query 3	(plant growth-promoting OR plant growth promotion) AND (zinc solubilizing bacteria OR zinc-solubilizing bacteria)	13	14	168	247

Three queries using different keywords were conducted on PubMed and PubMed Central databases at two different dates. The number of articles found is indicated for each search category.

### Screening the *Bacillus* strains for PGP traits

For all biochemical assays, *Bacillus* isolate suspensions were adjusted to an optical density (OD) of 1.0 at 600 nm, and 30 μl of suspension was used to inoculate all media plates, unless otherwise stated. Also, all assays were performed in triplicate. In cases where the *Bacillus* isolates produced high amounts of exopolysaccharides in LB broth, strains were alternatively grown in those media from which they were originally isolated from (either TSA, TY, R2A, or R2A + salt).

#### Solubilization of phosphate and zinc oxide

Isolates were tested for their ability to solubilize phosphate (P) using the Pikovskaya *et al*.^89^ protocol. Briefly, bacteria were grown on Pikovskya’s (PVK) Agar (M520, Himedia) plates at 28 °C for 48 hours. Plates were observed for zone of clearance around the bacterial inoculum to identify bacteria capable of solubilizing P.

Similarly, modified PVK Agar^90^ was used to screen isolates for their ability to solubilize zinc oxide (ZnO). Here too, plates were observed for zone of clearance around the bacterial inoculum to identify bacteria capable of solubilizing ZnO.

#### Production of indole acetic acid (IAA), ammonia (NH_3_) and siderophores

Production of IAA was assessed using the Brick *et al*.^91^ protocol. Briefly, bacteria were grown in LBTD4 medium in L-Tryptophan (2.5 mM) for 48 hours. Fully grown cultures were centrifuged at 10000 x g for 30 min. Subsequently, 1 ml of the supernatant was mixed with two drops of orthophosphoric acid and 2 ml of the Salkowski reagent, then incubated at room temperature for 30 min. Development of pink color indicates IAA production.

NH_3_ production was assessed using the Cappuccino and Sherman (1996)^92^ protocol. Briefly, 50 μl of bacterial suspension was inoculated in 5 ml of peptone broth and incubated at 28 °C for 48–72 hours. Subsequently, 250 μl Nessler’s reagent was added to each tube. Development of brown- orange color was used as an indicator of ammonia production.

Siderophore production was detected qualitatively using the Blue Agar CAS assay as described by Louden *et al*.^93^. Formation of an orange-yellowish zone of clearance was used as an indicator of siderophore production.

#### Antimicrobial activity

Antimicrobial activity against plant pathogens (including *P. syringae* DC3000 and two pathogenic fungal strains *Botrytis cinerea* and *Alternaria brassicicola*) were detected using dual culture assay. Briefly, the fungal strains were grown at 24 °C for 20–25 days on Potato Dextrose Agar (PDA) plate. Fungal disks were made using the back side of sterile 200μl pipette tips, and placed on new plates in one side and the selected *Bacillus* isolates were streaked at one corner in other side. The plates were incubated at 28 °C and checked at 24 h, 48 h and 72 h for formation of cleared zone as an indicator of a potential biocontrol agent. For the antibacterial assay, *P. syringae* DC3000 and *Bacillus* isolates suspensions were grown at 28 °C, 200 rpm, overnight on LB broth adjusted to an optical density (OD) of 1.0 at 600 nm. The spread plate technique was used to inoculate LB agar plates with 100 μl aliquots of each individual DC3000 culture, and then sterile disks were soaked with the *Bacillus* suspension and added to each inoculated plate. Formation of a zone of clearance was used as an indicator of a potential biocontrol agent.

### Screening *Bacillus* strains for their tolerance against abiotic stresses

Isolates were screened for their resilience against heat and salt stress. In brief, the *Bacillus* isolate suspensions were grown in LB broth and adjusted to an optical density (OD) of 1.0 at 600 nm, before being used to prepare serial dilutions (10^−1^–10^−5^). An aliquot of 30 µL of each dilution suspension and the non-diluted suspension was used to inoculate each LB plate used in the salt and heat stress tests. *Bacillus* isolates inoculated on LB agar plates were incubated at multiple temperatures 28 °C (control temperature), 37 °C, 42 °C and 50 °C) and incubated for 48 hours. These *Bacillus* isolates suspensions were also inoculated on LB agar plates containing various salt concentrations (0.5%, 1%, 1.5%, and 2% NaCl) that were incubated at 28 °C.

### Assessing the *Bacillus* strains on plant growth promotion

The effect of inoculating *Arabidopsis thaliana* Col-0 with each of the shortlisted strains (*B. subtilis* (PK5-26), *B. cereus* (PK6-15), *B. badius* (PK3-68), *B. circulans* (PK3-15; PK3-138; PK3-109), *B. subtilis* (PK3-9; PK1-2; PK1-3) and *B. circulans* (PK5-39)) was evaluated. For this assessment, bacterial cultures were grown overnight in LB broth at 28 °C at 220 rpm. Culture concentrations were then adjusted to an optical density (OD) of 0.2, 0.02 and 0.01 at 600 nm for each bacterial strain. Bacterial cultures were then pelleted and washed with 1⁄2 MS media to remove all bacterial media from the bacterial suspension. A final inoculum of 10 μl of washed bacterial suspension was inoculated to each of the 5-days-old seedlings. The control used for this method was non-inoculated seedlings.

Seeds of *Arabidopsis thaliana* Col-0 were surface sterilized, stratified on half-strength Murashige and Skoog Basal Salt Mixture pH 5.8, 0.9% agar (½MS) (Murashige and Skoog, 1962) without sucrose media for 2 days and then germinated for an additional 5 days at 22 °C, long day photoperiod (16 h light/8 h dark), 70 Lux. Germinated seedlings were then transferred to fresh ½MS and ½MS + 100 mM NaCl and inoculated with 10 µl of individual shortlisted strains (suspended in ½MS media adjusted to an optical density (OD) of 1.0 at 600 nm). Inoculated seedlings were incubated at 22 °C, 16 h light/8 h dark, 70 Lux, for a total of 15 days post-inoculation. At least three independent experiments corresponding to 36 plant seedlings were used for each strain analyzed. To clarify, six seedlings per plate = one technical replicate. In total, three technical replicates were analyzed per treatment (18 seedlings for non-salt conditions and 18 seedlings for salt stress conditions). On day 9, lateral root density was calculated using the standard calculation: The average number of lateral roots/cm of root length from 36 plants. Fresh weight was measured for plants growing in ½ MS on day 12 and for those growing in ½MS + 100 mM NaCl on day 15. The experiment was done in 3 biological replicates of 36 plants each for each treatment. Here it should be noted that since, roots of plants grown on non-salt media already reached the bottom of the plates after 12 days, we harvested the plants at this time point. In contrast, plants grown under salt stress is slow growing and hence were harvested at 15 days. It should be noted that we only compared salt to salt and non-salt to non-salt treated *Bacillus*-inoculated plants to each other.

Statistical differences were determined using the Mann-Whitney U Test (https://www.socscistatistics.com/tests/mannwhitney/Default2.aspx). For the comparison between non-salt controls and samples, and salt stressed controls and samples, the means were used. A *p* value of ≤0.05 was considered as statistically significant (indicated with asterisks in Fig. 4). Note that in this study we opted for the non-parametric Mann-Whitney U test.

## Supplementary information

Table S1

Table S2

Table S3

## Data Availability

All data used in this study have been included in this article and its Supplementary Files. The obtained 16S rRNA gene sequences were deposited in NCBI database and were assigned accession numbers MG988210 to MG988268.
