# Author Correction: Bioprospecting desert plant *Bacillus* endophytic strains for their potential to enhance plant stress tolerance

**DOI:** 10.1038/s41598-020-58957-w

**Published:** 2020-02-14

**Authors:** Ameerah Bokhari, Magbubah Essack, Feras F. Lafi, Cristina Andres-Barrao, Rewaa Jalal, Soha Alamoudi, Rozaimi Razali, Hanin Alzubaidy, Kausar H. Shah, Shahid Siddique, Vladimir B. Bajic, Heribert Hirt, Maged M. Saad

**Affiliations:** 1 King Abdullah University of Science and Technology (KAUST), Center for Desert Agriculture, Thuwal, 23955-6900 Kingdom of Saudi Arabia; 2 King Abdullah University of Science and Technology (KAUST), Computational Bioscience Research Center (CBRC), Thuwal, 23955-6900 Kingdom of Saudi Arabia; 3 King Abdulaziz University, Science and Arts College, Department of Biology, Rabigh, 21589 Kingdom of Saudi Arabia; 4 Bahauddin Zakariya University, Institute of Pure and Applied Biology, Multan, 60800 Pakistan; 5 UC Davis, Department of Entomology and Nematology, One Shields Avenue, Davis, USA; 6 Max F. Perutz Laboratories, University of Vienna, Dr. Bohrgasse 9, 1030 Vienna, Austria; 7 Present Address: Exploration and Petroleum Engineering Center - Advanced Research Center (EXPEC ARC), Saudi Aramco, Dhahran Saudi Arabia; 8 Present Address: Zayed University, College of Natural and Health Sciences, Abu-Dhabi, 144534 United Arab Emirates; 9 University of Jeddah, P-O-BOX No.80327, Jeddah, 21589 Saudi Arabia

Correction to: *Scientific Reports* 10.1038/s41598-019-54685-y, published online 03 December 2019

The original version of this Article omitted an affiliation for Rewaa Jalal. The correct affiliations for Rewaa Jalal are listed below:

King Abdullah University of Science and Technology (KAUST), Center for Desert Agriculture, Thuwal, 23955-6900, Kingdom of Saudi Arabia

University of Jeddah, P-O-BOX No.80327, Jeddah 21589, Saudi Arabia

This has now been corrected in the HTML and PDF versions of this Article, and in the accompanying Supplemental Material.

