# Supplementary material for: Bioprospecting desert plant Bacillus endophytic strains for their potential to enhance plant stress tolerance
Source: Sci Rep. 2019 Dec 3;9:18154. doi: 10.1038/s41598-019-54685-y (PMC6890672; doi:10.1038/s41598-019-54685-y)
Supplement: Supplementary file 1 — Table S1 [file 41598_2019_54685_MOESM1_ESM.docx]

# Bioprospecting desert plant *Bacillus* endophyte strains for their potential to enhance plant stress tolerance

Ameerah Bokhari ^1#^, Magbubah Essack ^2^, Feras F. Lafi ^1##^, Cristina Andres-Barrao ^1^, Rewaa Jalal ^1,7^, Soha Alamoudi ^3^, Rozaimi Razali ^2^, Hanin Alzubaidy ^1^, Kausar H. Shah ^4^, Shahid Siddique ^5^, Vladimir B. Bajic ^2^, Heribert Hirt ^*1,6^, Maged M. Saad ^1^

^1^ King Abdullah University of Science and Technology (KAUST), Center for Desert Agriculture, Thuwal 23955-6900, Kingdom of Saudi Arabia.

^2^ King Abdullah University of Science and Technology (KAUST), Computational Bioscience Research Center (CBRC), Thuwal 23955-6900, Kingdom of Saudi Arabia.

^3^ King Abdulaziz University, Science and Arts College, Department of Biology, Rabigh 21589, Kingdom of Saudi Arabia.

^4^ Bahauddin Zakariya University, Institute of Pure and Applied Biology, Multan 60800, Pakistan

^5^ UC Davis, Department of Entomology and Nematology, One Shields Avenue, USA

^6^Max F. Perutz Laboratories, University of Vienna, Dr. Bohrgasse 9, 1030 Vienna, Austria

^7^University of Jeddah, P-O-BOX No.80327, Jeddah 21589, Saudi Arabia

^#^ Present address: Exploration and Petroleum Engineering Center - Advanced Research Center (EXPEC ARC), Saudi Aramco, Dhahran, Saudi Arabia.

^##^ Present address: Zayed University, College of Natural and Health Sciences, Abu-Dhabi 144534, United Arab Emirates.

*Corresponding Author: heribert.hirt@kaust.edu.sa Tel.: [+966-544-700-088]; Fax: [+966-12-802-1344]

**AB:** ameerah.bokhari@kaust.edu.sa

**ME:** magbubah.essack@kaust.edu.sa

**FFL:** Feras.Lafi@zu.ac.ae

**CAB:** cristina.andresbarrao@kaust.edu.sa

**RJ:** rewaa.jalal@kaust.edu.sa

**SA:** soha.amoudi@kaust.edu.sa

**RR:** rozaimirazali@gmail.com

**HA:** [hanin.alzubaidy@kaust.edu.sa](mailto:hanin.alzubaidy@kaust.edu.sa)

**KHS:** kausarshah@bzu.edu.pk

**SS:** siddique@uni-bonn.de

**VBB:** vladimir.bajic@kaust.edu.sa

**HH:** heribert.hirt@kaust.edu.sa

**MMS:** maged.saad@kaust.edu.sa

### Table S1: Site description, physicochemical properties and elemental composition of soil*:* Data presented are mean values (± standard deviation) of 9 independent replicates.

| **Location** | **Pakistan (Thar desert)** |
| --- | --- |
| Latitude; Longitude | 24°45'00.4"N 69°56'00.8"E |
| Total precipitation per year (mm) | 50 |
| Maximum temperature (°C) | 36 |
| Average temperature (°C) | 30 |
| Minimum temperature (°C) | 18 |
| Soil color and texture | Brown, sandy soil |
| Soil pH | 7.31 |
| Soil Moisture content (%) | 5 |
| Nitrogen content (g Kg^-1^) | 0.27 ± 0.52 |
| Carbon content (g Kg^-1^) | 13.7 ± 1.10 |
| Element measurement |  |
| P (g Kg^-1^) | 0.43 ± 0.015 |
| K (g Kg^-1^) | 6.11 ± 1.14 |
| Ca (g Kg^-1^) | 68.14 ± 5.52 |
| Mg (g Kg^-1^) | 5.75 ± 0.22 |
| B (mg Kg^-1^) | 89.85 ± 6.84 |
| Cu (mg Kg^-1^) | 22.71 ± 2.21 |
| Mn (mg Kg^-1^) | 427.16 ± 20.54 |
| Zn (mg Kg^-1^) | 109.65 ± 17.21 |
| Na (mg Kg^-1^) | 379.2 ± 76.20 |
| Ni (mg Kg^-1^) | 52.63 ± 3.74 |
| S (mg Kg^-1^) | 338.48±38.5 |
| Pb (mg Kg^-1^) | 254.2 ± 50.97 |
