# Supplementary material for: Bioprospecting desert plant Bacillus endophytic strains for their potential to enhance plant stress tolerance
Source: Sci Rep. 2019 Dec 3;9:18154. doi: 10.1038/s41598-019-54685-y (PMC6890672; doi:10.1038/s41598-019-54685-y)
Supplement: Supplementary file 3 — Table S3 [file 41598_2019_54685_MOESM3_ESM.docx]

# Bioprospecting desert plant *Bacillus* endophyte strains for their potential to enhance plant stress tolerance

Ameerah Bokhari ^1#^, Magbubah Essack ^2^, Feras F. Lafi ^1##^, Cristina Andres-Barrao ^1^, Rewaa Jalal ^1,7^, Soha Alamoudi ^3^, Rozaimi Razali ^2^, Hanin Alzubaidy ^1^, Kausar H. Shah ^4^, Shahid Siddique ^5^, Vladimir B. Bajic ^2^, Heribert Hirt ^*1,6^, Maged M. Saad ^1^

^1^ King Abdullah University of Science and Technology (KAUST), Center for Desert Agriculture, Thuwal 23955-6900, Kingdom of Saudi Arabia.

^2^ King Abdullah University of Science and Technology (KAUST), Computational Bioscience Research Center (CBRC), Thuwal 23955-6900, Kingdom of Saudi Arabia.

^3^ King Abdulaziz University, Science and Arts College, Department of Biology, Rabigh 21589, Kingdom of Saudi Arabia.

^4^ Bahauddin Zakariya University, Institute of Pure and Applied Biology, Multan 60800, Pakistan

^5^ UC Davis, Department of Entomology and Nematology, One Shields Avenue, USA

^6^Max F. Perutz Laboratories, University of Vienna, Dr. Bohrgasse 9, 1030 Vienna, Austria

^7^University of Jeddah, P-O-BOX No.80327, Jeddah 21589, Saudi Arabia

^#^ Present address: Exploration and Petroleum Engineering Center - Advanced Research Center (EXPEC ARC), Saudi Aramco, Dhahran, Saudi Arabia.

^##^ Present address: Zayed University, College of Natural and Health Sciences, Abu-Dhabi 144534, United Arab Emirates.

*Corresponding Author: heribert.hirt@kaust.edu.sa Tel.: [+966-544-700-088]; Fax: [+966-12-802-1344]

**AB:** ameerah.bokhari@kaust.edu.sa

**ME:** magbubah.essack@kaust.edu.sa

**FFL:** Feras.Lafi@zu.ac.ae

**CAB:** cristina.andresbarrao@kaust.edu.sa

**RJ:** rewaa.jalal@kaust.edu.sa

**SA:** soha.amoudi@kaust.edu.sa

**RR:** rozaimirazali@gmail.com

**HA:** [hanin.alzubaidy@kaust.edu.sa](mailto:hanin.alzubaidy@kaust.edu.sa)

**KHS:** kausarshah@bzu.edu.pk

**SS:** siddique@uni-bonn.de

**VBB:** vladimir.bajic@kaust.edu.sa

**HH:** heribert.hirt@kaust.edu.sa

**MMS:** maged.saad@kaust.edu.sa

**Table S3**. *Bacillus* strains screened for their ability to confer plant growth promotion under salt stress conditions. The asterisk indicates that the dissimilarity between the control samples and the inoculated samples under the same conditions are statistically different based on Mann-Whitney U Test (*P < 0.05, **P < 0.01, ***P < 0.001). Bacteria were categorized as plant growth-promoting bacteria ((+)PGPB) and/or salt tolerance plant growth-promoting bacteria ((+)ST-PGPB) if they confer a statistically significant increase to *A. thaliana* fresh weight compared to the uninoculated control in the absence of NaCl and in the presence of NaCl, respectively.

| **Strain code** | **Identification based on 16S rRNA sequencing** | **Conferring PGP traits and salt tolerance** | **Production and Solubilization Capabilities** | **Bacterial Resilience** | |
| --- | --- | --- | --- | --- | --- |
|  |  |  |  | **Salinity**  **(M)** | **Heat**  **(^o^C)** |
| PK6-15 | *Bacillus cereus* | (-)PGPB  (+)ST-PGPB *** | Zinc & Ammonia | 1.5 | 42 |
| PK5-26 | *Bacillus subtilis* | (+)PGPB *** (+)ST-PGPB *** | Phosphate, Siderophore & Ammonia | 2 | 50 |
| PK3-109 | *Bacillus circulans* | (-)PGPB  (+)ST-PGPB *** | IAA & Exopolysaccharide | 1.5 | 42 |
| PK3-9 | *Bacillus subtilis* | (-)PGPB  (+)ST-PGPB *** | Siderophore & Ammonia & Exopolysaccharide | 1.5 | 50 |
| PK3-68 | *Bacillus badius* | (-)PGPB  (+)ST-PGPB *** | IAA &  Ammonia & Exopolysaccharide | 1.5 | 42 |
| PK3-15 | *Bacillus circulans* | (-)PGPB  (+)ST-PGPB ** | IAA | 1.5 | 42 |
| PK1-3 | *Bacillus subtilis PY79* | (-)PGPB  (+)ST-PGPB ** | Siderophore & Ammonia & Exopolysaccharide | 1.5 | 50 |
| PK1-2 | *Bacillus subtilis* | (-)PGPB  (+)ST-PGPB * | Siderophore & Ammonia | 1.5 | 50 |
| PK5-39 | *Bacillus circulans* | (-)PGPB  (-)ST-PGPB *** | Ammonia & Exopolysaccharide | 2 | 50 |
| PK3-138 | *Bacillus circulans* | (-)PGPB  (-)ST-PGPB ** | IAA | 1.5 | 50 |
